# 

**Published:** 1854-08

**Authors:** 


					﻿No. Ill ]
AUGUST.
[VOL.X.
buffalo
MEDICAL JOURNAL
ajtj»
lieutetv
or
MEDICAL AND SURGICAL SCIENCE.
AUSTIN FLINT, M. D.,	)
SANFORD B. HUNT, M. D., < Enm,TO-
BUFFALO, N. Y.
PUBLISHED BY JEWETT, THOMAS A CO
Commercial Advertiser Bulldtr.gn.
1*54.
Price 82.50 per atrun is aHvanee.
				

